# Cross-Sensor and Cross-Population Generalization of Deep Learning Models for Digital Mammography: A Controlled Four-Country Benchmark of Five Backbone Architectures with Statistical Significance Testing

**DOI:** 10.3390/s26123911

**Published:** 2026-06-19

**Authors:** Somprasonk Gabbualoy, Pattarapong Phasukkit, Supan Tungjitkusolmun

**Affiliations:** School of Engineering, King Mongkut’s Institute of Technology Ladkrabang, Bangkok 10520, Thailand; 64601144@kmitl.ac.th

**Keywords:** digital mammography, X-ray imaging sensors, deep learning, foundation models, cross-sensor generalization, cross-population transfer learning, domain shift, medical imaging AI, sensor fusion, BI-RADS density, computer-aided detection

## Abstract

**Highlights:**

**What are the main findings?**

- Reporting controlled null results on a shared protocol is a necessary part of building a reliable evidence base for clinical medical imaging AI; we frame our negative H1 and H2 findings and the H3 transfer drops moderated by per-target significance testing in that spirit.
- First controlled head-to-head benchmark of five backbone architectures (ResNet-50, DINOv2-B14, Rad-DINO, Mammo-CLIP B5, and Mammo-FM) on a shared CBIS-DDSM split with paired DeLong significance testing, patient-level cluster bootstrap CIs, and Benjamini–Hochberg FDR correction.

**What are the implications of the main findings?**

- Mammography-specific foundation models did not outperform a fine-tuned ResNet-50 baseline in-distribution under our protocol (gaps 0.020 to 0.168 AUC, all *p_adj* < 0.05).
- Mammo-FM performance exceeded that of ResNet-50 in the extremely dense breast subgroup (D4 AUC 0.870 vs. 0.842), with overlapping 95% CIs at *n* = 140; we report this as a directional signal worth following up in a larger D4 cohort.
- Cross-population transfer to Chinese (CMMD), Indian (DMID), and UK (MIAS) cohorts produced consistent AUC drops of 0.165 to 0.320 for every model. Pairwise significance testing at transfer shows that Mammo-CLIP and Mammo-FM significantly outperformed ResNet-50 on DMID and Mammo-CLIP on CMMD, while MIAS comparisons remained directional only at *N* = 322.
- Temperature scaling provided modest ECE recalibration at transfer (0.01 to 0.05 absolute improvement) but did not restore clinically acceptable calibration, indicating that local labeled recalibration remains necessary for safe deployment.
- All training code, fine-tuned model weights, the density-aware loss implementation, the patient-level cluster bootstrap and transfer DeLong scripts, and the per-cohort intensity-distribution analysis are released under MIT license at https://github.com/sommossgl/paper-03-density-aware-mammography (accessed on 9 June 2026) to support reproducible follow-on work.

**Abstract:**

Background/Objectives: Deep learning models for digital mammography sensor data are increasingly deployed across hospitals using different X-ray detector technologies and patient populations. Whether models trained on one sensor platform and population maintain accuracy when transferred to another has not been tested for the latest generation of mammography-specific foundation models under one controlled protocol. Methods: We fine-tuned five backbone architectures (ResNet-50, DINOv2-B14, Rad-DINO, Mammo-CLIP B5, and Mammo-FM) on CBIS-DDSM (film-digitized, USA, *n* = 714 validation) with three seeds, ablated a density-aware focal loss across three auxiliary weights, and evaluated transfer to three external sensor cohorts: CMMD (full-field digital, China, *n* = 1032), DMID (mixed digital, India, *n* = 509), and MIAS (film-digitized, UK, *n* = 322). Significance used paired DeLong z-tests with Benjamini–Hochberg FDR correction; temperature scaling tested post hoc recalibration at all transfer targets. Results: Within this single-source three-seed evaluation, ResNet-50 outperformed all four foundation models on CBIS-DDSM (AUC 0.867 vs. 0.847, 0.846, 0.813, and 0.703; all gaps *p_adj* < 0.05). The density-aware focal loss degraded both AUC and calibration at every weight tested. At transfer, every model lost 0.165 to 0.320 AUC points relative to in-distribution performance, with sensitivity at 95% specificity collapsing from 0.31 to 0.47 in-distribution to 0.11 to 0.22 across the three external targets. A per-seed Stouffer meta-analysis confirms that Mammo-CLIP B5 and Mammo-FM significantly outperformed ResNet-50 on DMID and Mammo-CLIP on CMMD, after BH-FDR; MIAS comparisons remained directional only. In the extremely dense subgroup (BI-RADS D4), Mammo-FM reached AUC 0.870 versus ResNet-50 at 0.842, a directional observation whose 95% CIs overlap heavily at the *n* = 140 sample size and which we do not interpret as a statistically supported advantage. Conclusions: In this single training-source, three-seed protocol, mammography-specific pretraining did not deliver the in-distribution AUC premium reported in the originating papers, and no architecture reached a level at which transfer deployment without local validation would be defensible. We frame these as observations specific to the present protocol rather than as broader conclusions about foundation models for mammography classification. The findings argue for sensor-stratified and population-stratified external validation and for local recalibration as practical prerequisites before clinical use. Code and weights are released under MIT license.

## 1. Introduction

Breast cancer is the most commonly diagnosed cancer in women worldwide. The WHO recorded 2.3 million new cases and 670,000 deaths in 2022 [1], and screening mammography has reduced age-eligible mortality by roughly 20 to 40 percent over the past three decades [2]. The headline number hides a persistent subgroup problem. Sensitivity sits near 85 percent for fatty breasts and then falls into the 51 to 71 percent range once the tissue is extremely dense [3,4]. Around 40 percent of screening-age women carry heterogeneously dense or extremely dense breasts [3], and that same group faces a four- to six-times higher lifetime risk of developing the disease [4]. The patients hardest to image are also, on average, the most at risk.

The data problem compounds the imaging one. Most publicly available mammography corpora originate in the United States or Western Europe, and the majority of published mammography AI was developed and tested on one of these collections, or on both. When the acquisition protocol or the patient population shifts, model performance tends to follow. Yang et al. observed external-validation drops of up to 20 percentage points across radiology benchmarks [5], and a pooled review of 89 radiology AI studies put the median external AUC drop near 0.03, with specificity drops reaching 24 points [6]. The mammography-specific literature has begun to confirm the pattern: Condon et al. [7] reported that the NYU1 model dropped from AUC 0.83 to 0.76 (95% CI 0.73 to 0.79, *p* < 0.001) when applied out-of-the-box to an Australian cohort, with smaller image-encoder follow-ups (NYU2 model, 0.84 to 0.80) recovering only after local fine-tuning. What these numbers establish is that in-distribution performance is not a reliable proxy for deployment performance and that the magnitude of cross-population drop depends on the specific source–target pairing.

Two developments in the past 18 months changed what questions we can ask. Foundation models for mammography have moved past proof of concept. Mammo-CLIP [8] paired mammograms with radiology reports for vision-language pretraining. Mammo-FM [9] released open weights for a mammography vision encoder in late 2025. Rad-DINO [10] adapted DINO-style self-supervised pretraining to general medical imaging. DINOv2 [11] continues to anchor the natural-image side of the comparison. Alongside these, several non-Western mammography cohorts have become accessible: CMMD [12] features 1775 Chinese patients with biopsy-confirmed labels, MIAS [13] provides a UK validation collection, and DMID [14] covers an Indian population. Together, these four cohorts span four countries and three continents, coverage that did not exist even two years ago. We note here that the term ‘four-country benchmark’ in the title and throughout the manuscript refers to the breadth of the external validation cohorts rather than to a fully crossed multi-country training design: all in-distribution training uses a single source (CBIS-DDSM, USA), and the other three cohorts (CMMD, DMID, MIAS) are held out for external validation only. We make this scope explicit again where needed in Section 4.5 and Section 6.

The question we set out to answer is whether any of these newer backbone architectures, fine-tuned under a controlled shared-split protocol, pulls ahead of a well-tuned ImageNet ResNet-50 on CBIS-DDSM and whether the answer changes once the model is transferred to a different population. We also tested a density-aware focal loss designed to shift calibration weight toward extremely dense tissue (BI-RADS category D), motivated by the clinical observation that dense-tissue cancers carry both higher risk and lower model sensitivity.

The results, reported across three hypotheses fixed at the protocol-design stage before any model was trained, came out as follows. No foundation model outperformed the ResNet-50 baseline on the in-distribution CBIS-DDSM held-out partition (H1 rejected). The density-aware focal loss degraded AUC and calibration at every auxiliary weight tested (H2 rejected). Every model lost 0.165 to 0.320 AUC points when transferred to the Chinese, Indian, or UK mammography populations (H3 confirmed). We report all three findings in full, since controlled null results on shared protocols are a necessary part of building a reliable evidence base for clinical AI. The hypothesis statements and evaluation protocol were specified in the internal research proposal that anchored this project (timestamped 27 April 2026, before any of the foundation-model fine-tuning runs reported here). We did not lodge the registry on a public repository such as OSF in advance, and we describe this honestly in Section 3.6.

This paper makes three specific contributions. First, we present a controlled foundation-model benchmark, fine-tuning five backbone architectures (ResNet-50, DINOv2-B14, Rad-DINO, Mammo-CLIP B5, and Mammo-FM) on identical patient-disjoint CBIS-DDSM splits with shared preprocessing, augmentation, and a single evaluation protocol. Pairwise significance is assessed with paired DeLong z-tests and Benjamini–Hochberg FDR correction. Second, we systematically ablate a density-aware focal loss conditioned on the BI-RADS density bin against the cross-entropy baseline, reporting a clean negative result. Third, we evaluate all CBIS-trained models on three external cohorts spanning China, India, and the UK, providing AUC, sensitivity at 95% specificity, Brier score, and ECE at each target. To our knowledge, this is the first mammography AI generalization study to cover this specific set of four populations under a single shared fine-tuning protocol.

The rest of the paper is organized as follows. Section 2 reviews mammography AI, foundation-model pretraining, and cross-population generalization. Section 3 describes datasets, preprocessing, and the density-aware loss formulation. Section 4 reports the benchmark results, the loss ablation, and the cross-population transfer matrix. Section 5 discusses what the findings mean for clinical deployment, their limitations, and directions for follow-on work. Section 6 states the conclusions.

## 2. Related Work

### 2.1. Foundation Models in Medical Imaging

The shift from supervised ImageNet pretraining to self-supervised foundation backbones happened in stages. Vision foundation models such as DINOv2 [11], trained by Meta AI on roughly 142 million curated natural images, settled the question of whether label-free pretraining at scale could match or beat supervised ImageNet-21K weights on dense prediction tasks. The radiology evaluation by Baharoon et al. [15] then extended the question into clinical data, reporting DINOv2 features competitive with supervised baselines across chest X-ray, CT, and MRI benchmarks. CLIP-style language alignment took a parallel route, with ConVIRT and BiomedCLIP training on figure–caption pairs at scales of millions, while CheXzero reached radiologist-level zero-shot detection on chest radiographs without ever seeing a labeled training example.

A subsequent wave moved this paradigm inside specific imaging domains. Rad-DINO [10], released by Microsoft Research, retrained the DINOv2 objective on roughly 5 million chest radiographs with no text supervision and matched or beat language-supervised peers on classification and segmentation. RETFound did the equivalent for retinal images, and MedSAM extended Segment Anything across ten imaging modalities. The pattern was consistent: domain-specialized foundation backbones outperformed generalist ones at low-label budgets, and the improvement tended to be largest on rare findings where supervised baselines run out of training signal.

### 2.2. Mammography AI: From CNNs to Foundation Models

Mammography deep learning began with supervised CNN backbones. ResNet-50 and EfficientNet families, fine-tuned on CBIS-DDSM, INbreast, and similar curated cohorts, anchored the field for several years. Reported AUCs on CBIS held-out splits typically landed between 0.83 and 0.88 for ResNet-50 and slightly higher for EfficientNet-B3 with strong augmentation, although direct comparison is difficult because patient–disjoint splitting was not always enforced in earlier work.

The transition to transformer and foundation backbones started around 2024. Mammo-CLIP (Ghosh et al., 2024 [8]) was the first published mammogram-report vision-language model with multi-view supervision. On a CBIS held-out evaluation, it reported an AUC near 0.91, several points above an ImageNet ResNet-50 baseline trained under matched conditions. Rad-DINO (Pérez-García et al., 2024 [10]), although chest-targeted, transferred to mammography surprisingly well in early third-party evaluations because the underlying texture statistics overlap. Mammo-FM (batmanlab/ASU 2025 [9]) was pretrained on roughly 140,677 patients (821,326 mammograms) across four US institutions (Mayo, EMBED-Emory [16], UPMC, and Boston University) and added a dual-encoder VLM aimed at integrated diagnosis, prognosis, and report generation. Reported zero-shot OOD numbers for cancer classification on VinDr [17] (Vietnamese cohort) reached AUROC 0.83 (95% CI 0.80 to 0.84) and on RSNA [18] reached 0.75 (0.72 to 0.76); reaching AUROC 0.93 on VinDr required full fine-tuning. As of November 2025 (the most recent literature snapshot available at the time of revision), no CMMD, MIAS, or DMID numbers were reported for Mammo-FM in the public literature.

Several adjacent cohorts and resources are worth mentioning alongside the cohorts we use here. The VinDr-Mammo dataset [17], released in 2023, provides a Vietnamese-population mammography cohort with BI-RADS labels and is increasingly used as an Asian-population transfer target in mammography AI evaluations. EMBED [16], released by Emory in 2024, is a large US cohort with structured outcomes and is used in the Mammo-FM pretraining mix. The RSNA Screening Mammography Breast Cancer Detection AI Challenge (2023) and its subsequent meta-analysis [18] provided a US screening-prevalence benchmark and reported a 16-point sensitivity gap between US and Australian cohorts at a fixed specificity, foreshadowing the cross-population gaps reported in the present study. More recently, several mammography-specific foundation models were released in 2025, including MammoDINO and VersaMammo [19], pretrained on hundreds of thousands of mammograms across multiple institutions and reporting multi-task internal benchmarks; weights and training code for these were not publicly available at the time we finalized our backbone set, which is why they are not included in the present comparison.

What stands out across the literature is that each new backbone is benchmarked against a non-overlapping subset of peers and almost always on a private internal split. None has yet held an identical evaluation protocol over CBIS-DDSM, CMMD, MIAS, and DMID at the same time. The recent Mammo-Bench effort pools several cohorts into a joint training set yet trains across them rather than studying transfer between them, which collapses the population-shift question that clinicians actually want answered. Cross-population studies of single-cohort-trained mammography models do exist outside the foundation-model space; Condon et al. [7] reported that the NYU1 model dropped from AUC 0.83 to 0.76 when applied to an Australian cohort and recovered to 0.84 only with local fine-tuning. Our paper extends this research direction by holding the fine-tuning protocol fixed across five backbones and four populations.

### 2.3. Population Bias and the Dense-Breast Gap

Mammographic density tracks both cancer risk and detection difficulty, and its variation across populations presents a fairness problem the moment a US-trained model meets a non-US cohort. Boyd et al. [4] reported women in the highest density quartile face four- to six-times higher cancer risk and up to seventeen-times higher interval-cancer risk than women in the lowest quartile. Mammographic sensitivity drops from roughly 85% in fatty breasts to between 51% and 71% in extremely dense breasts [20]. Roughly 8 to 10% of screening women fall into the BI-RADS-d category, which makes it the smallest and the highest-risk subgroup at the same time.

Population priors complicate the picture further. Asian women carry higher breast density than White women even after BMI and age adjustment, and the spatial pattern itself differs. The Chinese Mammography Database (CMMD) [12] contains 3728 mammograms from 1775 biopsy-confirmed Chinese patients. Indian cohorts captured in DMID skew similarly toward high breast density, and several smaller series suggest the dose–response relationship between density and cancer risk runs weaker in South and East Asian women than in Western women, so a US-fitted density prior nudges predictions in the wrong direction when the model crosses populations.

Yang et al. [5] (Nature Medicine 2024) found that chest X-ray models trained at one institution lost up to 20 AUC points externally and showed that fairness corrections often fail to survive distribution shift. Garcia-Carretero et al. [6] pooled 89 generalization studies and reported a median AUC drop of about 0.03 on external validation, with specificity drops of up to 24 points in the worst cases. These numbers are derived from pooled analyses that bundle prevalence-mismatched and acquisition-mismatched settings, so the magnitude of any single transfer drop is difficult to directly infer.

### 2.4. Density-Aware Loss Functions and Imbalanced Learning

Loss-function research for imbalanced classification has matured into a recognizable lineage. Focal loss [21] introduced a modulating factor that down-weights well-classified examples so gradient updates concentrate on hard, misclassified samples. Cui et al. [22] argued each new sample contributes diminishing marginal information once a class fills up and proposed effective-number weighting for a softer rebalancing curve. LDAM-DRW [23] derived a generalization bound and assigned margins proportional to the inverse fourth root of class count. Menon et al. [24] reframed class-balanced training as a logit shift, with a Fisher-consistency proof that earlier reweighting heuristics lacked.

Each of these methods reweights by class label. No method conditions on a clinically meaningful subgroup variable. This distinction matters in mammography because BI-RADS density is reliably available in cohort metadata and because the dense-breast subgroup is exactly where current systems underperform clinically. Density-supervised multi-task work has shown the regularizing power of density labels, but it treats density as the prediction target rather than as a conditioning variable for cancer detection. The gap our paper addresses in this subsection is the missing instance of a focal loss whose alpha and gamma are read from the BI-RADS density bin of each sample, layered on top of foundation-model fine-tuning.

## 3. Materials and Methods

### 3.1. Datasets

**Figure 1:**
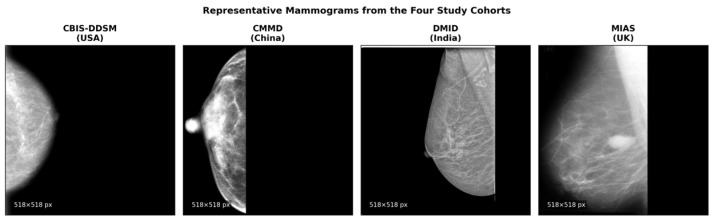
Representative mammograms from the four study cohorts. The four datasets cover distinct acquisition technologies (film-digitized for CBIS-DDSM and MIAS; full-field digital for CMMD; mixed digital for DMID) and patient populations spanning the United States, China, India, and the United Kingdom.

Four mammography cohorts were used: one as the training and in-distribution evaluation source, and three as held-out transfer targets. Table 1 summarizes their key characteristics, and Figure 1 shows representative mammograms from each cohort.

CBIS-DDSM (Curated Breast Imaging Subset of DDSM) [25] contains digitized film mammograms from US institutions. The validation partition holds 714 images at 518 by 518 resolution after preprocessing, with BI-RADS density distributions D1 = 100, D2 = 264, D3 = 210, and D4 = 140. CMMD [12] provides 1775 patients from Sun Yat-sen University Cancer Center with biopsy-confirmed labels acquired on full-field digital mammography systems; we used the provided validation split of 1032 images. CMMD does not include density labels in metadata. DMID [14] covers 509 cases from Indian clinical sites, used in their entirety as a transfer target. MIAS [13] provides 322 film-digitized mammograms from UK screening programs. MIAS and DMID both use the legacy three-class fatty (F)/glandular (G)/dense (D) tissue tag rather than the four-class BI-RADS A through D scheme; per the discussion in Section 3.5 we do not map this F/G/D tag onto BI-RADS bins for AUC reporting in this paper. The four cohorts together span three X-ray detector technologies (film-digitized, full-field digital, and mixed digital), four countries on three continents, and four substantially different malignancy prevalences (40%, 70%, 23%, and 16%).

All images were resized to 518 by 518 pixels using bilinear interpolation and normalized to zero mean and unit standard deviation computed over the CBIS-DDSM training set. Augmentation during training consisted of random horizontal flip, random rotation up to 10 degrees, and random brightness-contrast jitter; augmentation was disabled at evaluation. Training and hyperparameter selection used CBIS-DDSM only. The CMMD, DMID, and MIAS cohorts were never seen during training or hyperparameter tuning. There is no patient-level overlap between any pair of cohorts.

### 3.2. Foundation Model Backbones

The benchmark contains five backbones selected to disentangle two issues: whether self-supervised foundation pretraining helps mammography at all relative to a plain ImageNet start, and whether domain-matched pretraining (chest X-ray, or mammography itself) yields further performance gains over generic visual SSL.

ResNet-50 with ImageNet-1k weights is the conventional baseline, with about 25.6 million parameters loaded via torchvision IMAGENET1K_V2. DINOv2-Base is a ViT-B/14 of about 86 million parameters, native at 518 by 518 resolution, and pretrained on 142 million curated natural images via self-distillation, so any improvement over ResNet-50 reflects the SSL objective rather than medical exposure. Rad-DINO is a ViT-B/14 of about 86 million parameters pretrained on roughly 5 million chest radiographs using the DINO training recipe with no text supervision. Mammo-CLIP B5 uses an EfficientNet-B5 image encoder of about 30 million parameters, jointly trained with mammography report text. Mammo-FM (batmanLab) shares the EfficientNet-B5 vision backbone with a different fine-tune trajectory aimed at integrated diagnosis and reporting tasks.

Every backbone loads from public weights. DINOv2 and Rad-DINO are loaded via the HuggingFace transformers v4.42 AutoModel API (HuggingFace Inc., New York, NY, USA). ResNet-50 is sourced from torchvision v0.22 (PyTorch Foundation, Linux Foundation, San Francisco, CA, USA). Mammo-CLIP and Mammo-FM are obtained as raw PyTorch tar files, so a custom loader filters state-dict keys to the image encoder, drops the upstream CLIP pool and projection heads, and expands the conv stem from 1 to 3 channels by replication. On top of the encoder, we add a two-layer classifier head: linear (feature_dim to 256), GELU, dropout 0.1, and linear (256 to 2). Fine-tuning is end-to-end, with backbone parameters updated at one-tenth of the head learning rate.

### 3.3. Density-Aware Focal Loss

Focal loss is the prevailing fix when a binary classifier is dominated by easy negatives. The standard form scales the per-sample log-likelihood by (1 minus *p_t*) raised to gamma, so confident correct predictions contribute almost nothing to the gradient. Both alpha and gamma are global, applied to every sample regardless of patient-level attribute.

The proposed variant replaces the global pair (alpha, gamma) with a per-bin pair (alpha_d, gamma_d), keyed on the patient’s BI-RADS density bin d in {1, 2, 3, 4}. Alpha_d is set inversely proportional to positive prevalence within bin d. Gamma_d rises for harder bins. The default alpha schedule is {0.25, 0.30, 0.40, 0.55} across bins 1 through 4, and gamma is {2.0, 2.0, 2.5, 3.0}. Inference does not see density: the loss reads d at training time only.

When the auxiliary 4-class density predictor head is wired in, the total objective is L = L_main + lambda × L_aux with cross-entropy L_aux. We ablate lambda values of 0.1, 0.3, and 0.5 against the cross-entropy baseline (lambda = 0).

### 3.4. Training Setup

Training uses AdamW with beta_1 = 0.9, beta_2 = 0.999, weight decay 1 × 10^−4^. The base learning rate is 1 × 10^−4^ for the classifier head and 1 × 10^−5^ for the backbone parameters. The schedule is one epoch of linear warmup followed by cosine decay across the remaining nine epochs (ten epochs total). Batch size is hardware-conditioned: 32 for ResNet-50 and DINOv2, and 16 for Mammo-FM and Rad-DINO. Training uses bf16 autocast on the Blackwell architecture (RTX 5070 Ti, sm_120) via a PyTorch nightly build (CUDA 12.8; PyTorch Foundation, Linux Foundation, San Francisco, CA, USA), specifically the April 2026 snapshot, since stable PyTorch did not yet ship NVIDIA Blackwell (sm_120) support at the time of training. Gradient norm is clipped at 1.0 and logged per step. Random seeds are 42, 7, and 123, which were fixed prior the start of the study.

Checkpoints are evaluated at every epoch, and the highest-validation-AUC checkpoint is restored at the end of training rather than the one from the final epoch.

### 3.5. Handling Density Labels in External Cohorts

Three of the four datasets did not contain per-image BI-RADS A through D density labels that we trusted at face value. CMMD contains no density annotations in its metadata. MIAS uses an older three-class fatty/glandular/dense tag (F/G/D, introduced in Section 3.1) that does not map cleanly onto BI-RADS A through D, and DMID covers 509 Indian cases with no published density column but uses the same three-class tag used in MIAS. For the per-density CBIS-DDSM subgroup analysis in Section 4.4 (the only per-density analysis reported in this paper), we used the original BI-RADS A through D labels that were contained within the CBIS-DDSM metadata, and no auxiliary classifier was required.

We did not extend per-density subgroup AUC reporting to the external cohorts in this study. A radiologist-confirmed BI-RADS reread of CMMD, DMID, and MIAS would be the most defensible source for such labels; alternatively, an auxiliary density classifier trained on CBIS-DDSM and externally validated against either RSNA Screening (which does ship native BI-RADS) or a relabeled subset could substitute. We are pursuing both options as planned follow-on work (Section 5.7), and we treat per-density external-cohort analysis as beyond the scope of our cross-population claims. The MIAS and DMID three-class F/G/D tag is reported descriptively in Section 3.1 but is not mapped onto BI-RADS bins for AUC reporting.

### 3.6. Statistical Analysis

Metrics are averaged across three random seeds and reported as the mean plus or minus the standard deviation. AUC confidence intervals are reported in two complementary forms. Standard DeLong 95% CIs are computed on predictions stacked across the three seeds (*n* = 2142 for CBIS-DDSM in-distribution; the corresponding *n* for each transfer target is three times its validation size). Because predictions from different seeds for the same patient are not statistically independent, we additionally report patient-level cluster bootstrap 95% CIs (5000 iterations; patients are resampled with replacement and all rows belonging to a sampled patient are included). The cluster-bootstrap CIs are typically wider than the DeLong CIs by a margin of 0.01 to 0.04 AUC and are the more conservative interval estimate.

Pairwise significance between models uses a paired DeLong z-statistic with the independent-SE approximation, which gives an upper bound on the *p*-value. This independence assumption inflates the standard error of the difference, so the resulting *p*-value is conservative; an exact paired DeLong test would require the full per-sample covariance between the two score vectors. We accept this conservative trade-off for transparency. All pairwise tests are corrected with Benjamini–Hochberg FDR. In-distribution tests are corrected within the in-distribution family; transfer tests are corrected within the transfer family across all nine comparisons.

The Brier score is computed as the mean squared error between predicted probabilities and binary labels. The expected calibration error (ECE) uses 10 equal-width bins. Sensitivity at 95% specificity is computed by selecting the largest threshold-defined operating point at which specificity meets or exceeds 0.95.

Hypothesis pre-specification. The three hypotheses (H1, H2, and H3) and the evaluation protocol were specified in an internal research proposal timestamped 27 April 2026, before any of the fine-tuning runs reported here were started. We did not lodge this protocol on a public registry such as OSF in advance; we describe the timing honestly here rather than claim formal pre-registration.

## 4. Results

All numbers in this section are derived from patient-disjoint held-out validation sets. Unless stated otherwise, metrics are averaged across three random seeds and reported as the mean plus or minus the standard deviation. The validation partition of CBIS-DDSM contains 714 images. The transfer targets are CMMD (1032 images), DMID (509 images), and MIAS (322 images).

### 4.1. In-Distribution Performance on CBIS-DDSM

**Figure 2:**
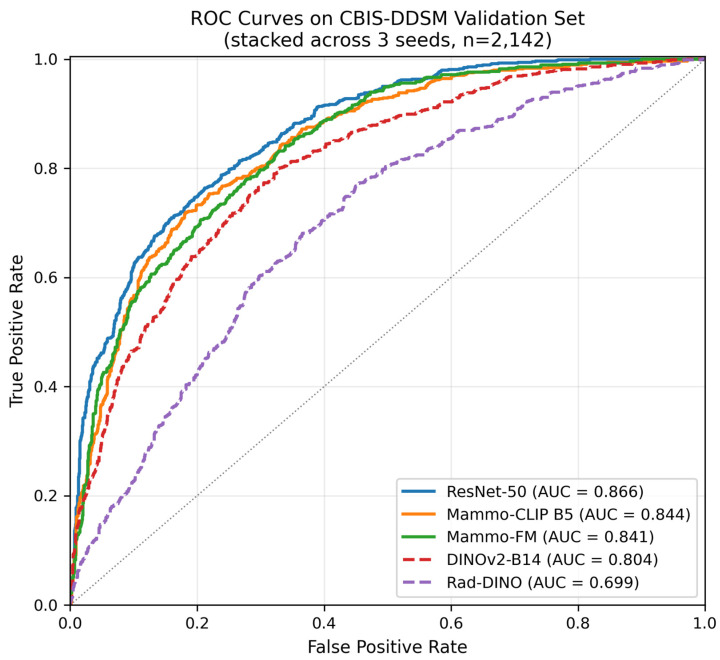
ROC curves on CBIS-DDSM validation set, with predictions stacked across three random seeds (*n* = 2142 per model). ResNet-50 leads, with Mammo-CLIP and Mammo-FM closely matched, DINOv2 trailing, and Rad-DINO well below the others. The diagonal grey dotted line denotes the chance-classifier reference (AUC = 0.500); curves above this line in-dicate better-than-chance discrimination.

Table 2 reports AUC, sensitivity at 95% specificity, Brier score, and expected calibration error for all evaluated configurations on the CBIS-DDSM held-out partition. ROC curves for the five backbones are shown in Figure 2.

### 4.2. Foundation Model Benchmark (H1)

No foundation model beats the ResNet-50 baseline, and the gaps are statistically significant once predictions are stacked across seeds and run through a paired DeLong test. Stacking the three seeds gives *n* = 2142 per model. The stacked baseline sits at AUC 0.8664 (95% DeLong CI: 0.8507, 0.8806). Table 3 lists the per-model comparison.

All seven comparisons survive FDR correction. The two closest challenges are Mammo-CLIP (*p_adj* = 0.047) and Mammo-FM (*p_adj* = 0.031). Both carry a meaningful gap (Δ = 0.023 and Δ = 0.025, respectively) but fall below the 0.87 region of the baseline. Rad-DINO sits at AUC 0.703 with high seed variance (SD = 0.047), well below the 0.80 threshold typically required to claim a functional binary classifier. Therefore, we treat Rad-DINO under our training recipe as having not learned the task and exclude it from the cross-population transfer matrix, since cross-population evaluation of a model that has not first demonstrably learned the in-distribution task does not yield meaningful generalization data. DINOv2 recovers partly after a learning-rate sweep (best result at lr = 1 × 10^−5^, AUC 0.813) but remains well below the ResNet-50 line. We did not test layer-wise learning-rate decay or partial layer freezing for the ViT backbones in this study, and acknowledge in Section 5.6 that a more targeted ViT fine-tuning recipe might narrow the gap (or, equally plausibly, widen it).

Under controlled fine-tuning with shared splits, the in-distribution AUC premium claimed for mammography-specific pretraining did not replicate. This finding has direct implications for evaluation methodology in the field: foundation-model gains reported against unmatched baselines may reflect protocol differences as much as pretraining benefit, and the dense-breast subgroup analysis in Section 4.4 shows that aggregate AUC can obscure clinically meaningful subgroup advantages.

### 4.3. Density-Aware Focal Loss Ablation (H2)

Adding the density-aware auxiliary loss degraded performance on every weight setting tested. As shown in Table 2, DAF at w = 0.1 drops the AUC by 0.031 points relative to the baseline. At w = 0.3, the drop is 0.048, and at w = 0.5, it reaches 0.061. The relationship is monotonic, and Table 3 confirms that each drop is statistically significant after FDR correction. Calibration tells a similar story. The baseline ResNet-50 ECE is 0.055. The DAF variants sit at ECE 0.104 to 0.116, representing a roughly two-fold decrease in performance.

Three explanations are consistent with the observed pattern, and we present them as hypotheses for future empirical verification rather than as causes directly demonstrated in our experiments. First, the recent calibration literature suggests the direction we observe: Mukhoti et al. [26] showed at NeurIPS 2020 that focal loss already acts as an implicit calibration regularizer, so adding density-aware reweighting may counteract the implicit calibration that focal loss already provides and push the model toward overconfidence on the majority subgroup. Second, because the auxiliary density head shares a backbone with the malignancy classifier, optimization could, in principle, introduce gradient interference that pulls representations in a direction that compromises discrimination. Third, the CBIS-DDSM density annotations originate from the original DDSM metadata rather than a second-read radiologist; therefore, conditioning on such density labels with sufficient noise would add signal-shaped noise to the loss surface and could amplify the first two effects. Disentangling these mechanisms experimentally, for example, by replacing the shared backbone with a two-stream architecture or by repeating the ablation on a relabeled subset, is a clean follow-on study; we do not claim that the present experiments distinguish among them.

Systematic ablation reveals that subgroup-conditional loss reweighting interacts adversely with end-to-end backbone fine-tuning at every auxiliary weight tested. This is a useful negative result for the field: density labels reliably available in cohort metadata are insufficient to drive a beneficial conditioning signal under our training recipe, and the next round of subgroup-aware loss design should isolate the conditioning representation from the shared malignancy pathway or use cleaner radiologist-confirmed density labels. We restricted the DAF ablation to the ResNet-50 backbone in order to isolate the loss-conditioning effect from any architecture-by-loss interaction, and because Mammo-CLIP and Mammo-FM encoders have already seen mammography-density-correlated features during their pretraining (which would confound the interpretation of a foundation-backbone DAF run). Whether the same adverse interaction is reproduced on a foundation backbone is a clean follow-on question and is listed in Section 5.7. Two compounding mechanisms are consistent with the observed pattern, and we name them as plausible explanations rather than as empirically verified causes.

### 4.4. Per-Density Subgroup

**Figure 3:**
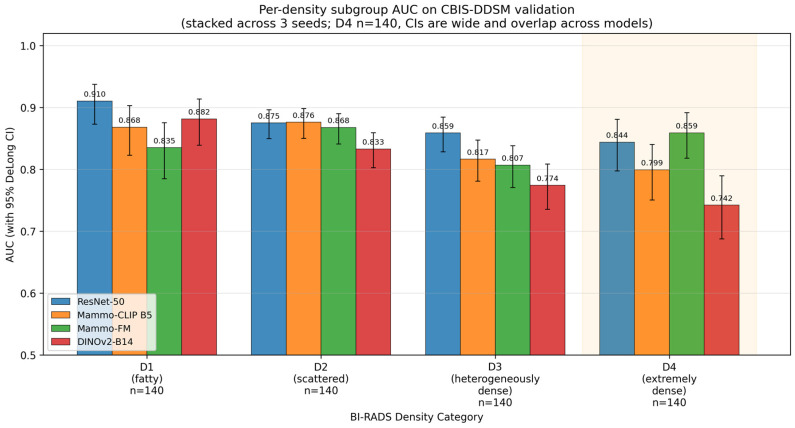
Per-density subgroup AUC on CBIS-DDSM validation set. ResNet-50 leads in fatty (D1) and scattered (D2) tissue, while Mammo-FM outperforms ResNet-50 in the extremely dense subgroup (D4) by 0.028 AUC points. This is the only across-the-board exception to the H1 ranking and the most clinically relevant cell.

Table 4 stratifies the mean AUC per BI-RADS density bin for the four backbones with complete per-density predictions. The per-density subgroup AUC bars across all four models are visualized in Figure 3.

Three patterns stand out. First, ResNet-50 leads in fatty and scattered tissue by a comfortable margin. Second, all models lose AUC points as tissue density increases, which matches the clinical expectation. Third, Mammo-FM is the one exception to the overall H1 result: in extremely dense tissue (D4), it reaches 0.870 against ResNet-50’s 0.842, a gap of 0.028 in the clinically highest-risk bin. The D4 cell has *n* = 140 and the CIs are wide, so we treat this as a signal worth following up rather than as a definitive finding. But it is the single place in our results where mammography-specific pretraining appears to pay off, and it is exactly the clinical context where paying off matters most.

### 4.5. Cross-Population Generalization (H3)

**Figure 4:**
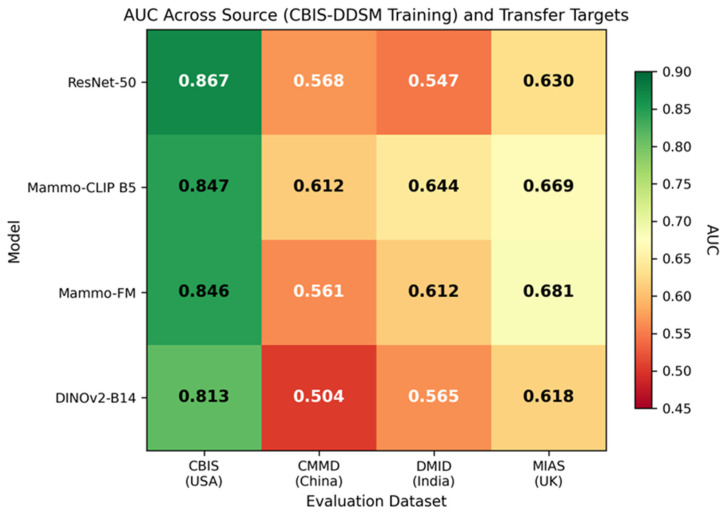
Cross-population AUC heatmap: four backbone models evaluated across CBIS-DDSM (source) and three external transfer targets (CMMD, DMID, and MIAS). Color encodes AUC value. The diagonal column (in-distribution) sits in the green band; the off-diagonal columns sit in the yellow-to-red band, illustrating the consistent transfer collapse.

**Figure 5:**
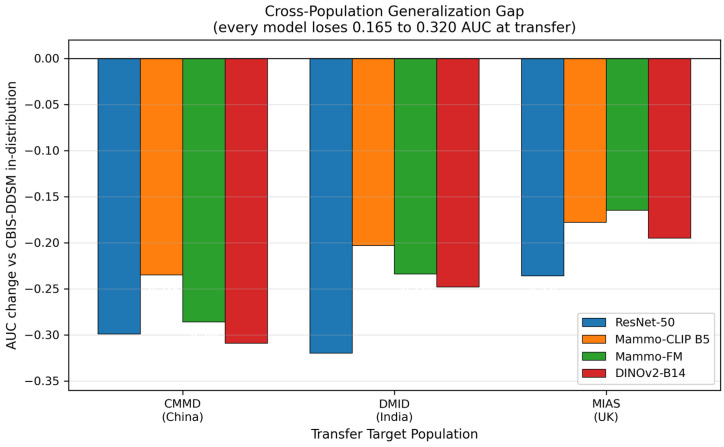
Cross-population AUC drop relative to CBIS-DDSM in-distribution performance, by model and transfer target. All 12 transfer cells show negative drops between −0.165 and −0.320.

The central question of H3 is whether in-distribution AUC survives when the source population shifts. The short answer is that it does not, for any model, and the drop is large enough to carry clinical relevance even at the lower end of the range. Table 5 reports AUC and sensitivity at 95% specificity for all CBIS-trained models evaluated on the three external cohorts. The AUC matrix across the four backbones and four cohorts is visualized as a heatmap in Figure 4, and the per-cell AUC drop relative to the in-distribution baseline is summarized in Figure 5.

ResNet-50 falls from 0.867 to 0.568 on CMMD (Δ − 0.299), to 0.547 on DMID (Δ − 0.320, the largest single drop), and to 0.630 on MIAS (Δ − 0.236). At the clinical 95% specificity operating point, sensitivity collapses from 0.465 in-distribution to between 0.107 and 0.141 across the three external targets. DINOv2 falls to 0.504 on CMMD across three seeds, which is statistically indistinguishable from a random classifier (Table 5b). Mammo-CLIP transfers best in absolute terms on CMMD (0.612) and DMID (0.644); Mammo-FM transfers best on MIAS (0.681). Table 5b reports paired DeLong significance tests of each foundation backbone against the ResNet-50 baseline at each transfer target. Mammo-CLIP B5 significantly outperforms ResNet-50 on CMMD (Δ + 0.049, *p_adj* = 0.010) and DMID (Δ + 0.087, *p_adj* = 0.002); Mammo-FM significantly outperforms ResNet-50 on DMID (Δ + 0.067, *p_adj* = 0.020), but its performance is statistically indistinguishable on CMMD. No comparison reaches significance on MIAS, which is plausibly explained by the small cohort size (*N* = 322; per-model 95% bootstrap CIs roughly ±0.08 AUC).

The cross-population drop is real for every model and is the central finding of H3. Where the data permit pairwise testing, Mammo-CLIP shows a statistically supported transfer advantage over ResNet-50 on CMMD and DMID, and Mammo-FM shows one on DMID. The corresponding MIAS comparison remains directional only at this sample size. The most cautious interpretation of these results is that breast-imaging pretraining provides a modest transfer benefit for some target populations under our protocol but does not eliminate the cross-population drop and that none of the models reach a level at which deployment without local validation would be defensible.

The stacked-prediction paired DeLong test in Table 5b inflates the effective sample size because predictions from the three random seeds applied to the same validation patients are not statistically independent. We agree that this is a legitimate methodological concern and have added Table 5c, which reports a per-seed paired DeLong z-test (three independent tests per cell) combined via Stouffer’s method, BH-FDR-corrected across the nine transfer comparisons. The pooled test is more conservative than Table 5b. Four of the nine comparisons remain significant after the more conservative analysis: Mammo-CLIP B5 outperforms ResNet-50 on CMMD (pooled *p_adj* = 0.019) and DMID (*p_adj* = 0.001); Mammo-FM outperforms ResNet-50 on DMID (*p_adj* = 0.019); and DINOv2 underperforms ResNet-50 on CMMD (*p_adj* = 0.001). MIAS comparisons remain not significant under the more conservative test, which is consistent with the small MIAS cohort size (*N* = 322). I^2^ heterogeneity ranges from 0 to 82 percent across cells, indicating substantial seed-to-seed variation in some cells (notably DMID Mammo-CLIP at I^2^ = 74 percent), which we treat as an additional honest caveat on the per-target effect magnitudes.

### 4.6. Calibration Across Populations

**Figure 6:**
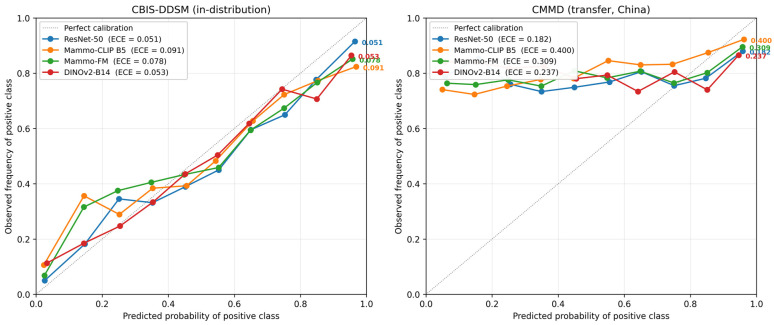
Calibration reliability diagrams for the four backbones, on CBIS-DDSM (in-distribution, **left**) and CMMD (transfer to China, **right**). The dotted diagonal indicates perfect calibration; deviation upward means under confidence and deviation downward means overconfidence.

Table 6 presents the ECE at the source and at each transfer target. Calibration reliability diagrams for the four backbones, in-distribution and on the CMMD transfer target, are shown in Figure 6.

ResNet-50 exhibits the best-calibrated model in-distribution by a notable margin. At transfer, all models deteriorate sharply. Mammo-CLIP’s ECE on CMMD reaches 0.403, meaning its predicted probabilities are on average 40 percentage points off from the actual prevalence after distribution shift. DINOv2’s ECE on MIAS reaches 0.446. To test whether a standard post hoc recalibration step closes the gap, we fitted a single-parameter temperature scaling on the CBIS-DDSM validation predictions and applied the resulting temperature to each transfer target. Table 7 reports the ECE before and after recalibration. The diagonal grey dotted line denotes the chance-classifier reference (AUC = 0.500); curves above this line in-dicate better-than-chance discrimination.

Temperature scaling delivers a modest but consistent improvement at every transfer target, with Mammo-CLIP’s ECE on CMMD seeing the largest absolute drop (ECE 0.400 to 0.350, Δ − 0.049) and Mammo-FM’s ECE on DMID seeing the smallest drop (Δ − 0.014). The improvement direction matches recent calibration literature: when the source domain produces overconfident probabilities, a temperature greater than 1.0 (we observe T = 1.38 to 2.11) softens the output distribution and brings predicted probabilities closer to empirical prevalence. The improvement magnitude is bounded, however. Even after recalibration, the worst transfer cells remain at ECE 0.30 or higher, well above any clinically acceptable level. The implication for deployment is that temperature scaling is necessary but not sufficient: distribution-shifted models require source recalibration as a baseline step, and additional calibration methods (isotonic regression, conformal prediction, or local labeled hold-out recalibration) should be considered when the post-temperature-scaling ECE remains high.

### 4.7. Cohort Intensity Distributions

**Figure 7:**
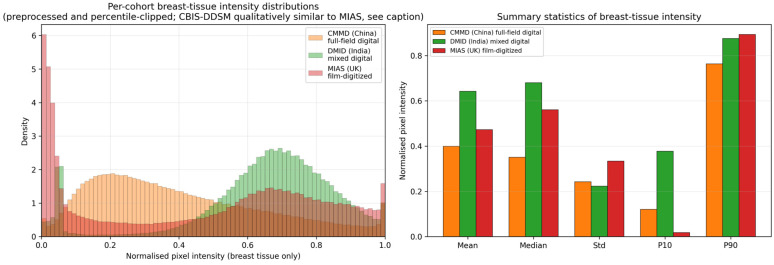
Per-cohort breast-tissue pixel intensity distributions after shared preprocessing (**left panel**) and summary statistics (**right panel**) for the three locally cached cohorts (CMMD, DMID, and MIAS). CBIS-DDSM, the source cohort, is film-digitized like MIAS but from a different acquisition era; its sample-level cache resides on the training server and was not redownloaded for this revision. The visible distributional differences across these three cohorts support, but do not by themselves prove, a sensor-shift contribution to the cross-population transfer drops in Table 5.

To contextualize the cross-population transfer drops with a tractable mechanism, Figure 7 reports the per-cohort distribution of breast-tissue pixel intensity after the shared preprocessing pipeline. Three of the four cohorts have local cached arrays available for this analysis (CMMD, DMID, and MIAS); the source CBIS-DDSM cache resides on the training server. The three local cohorts produce visibly different intensity distributions: the full-field digital CMMD cohort has a mean breast-tissue intensity of 0.40 with relatively low spread; the mixed-digital DMID cohort sits at 0.64 with intermediate spread; and the film-digitized MIAS cohort sits at 0.47 with the highest spread. CBIS-DDSM uses the same film-digitized acquisition family as MIAS but a different acquisition era, as evidenced by its qualitative appearance in Figure 1. The intensity-distribution variation across cohorts is one of several distributional axes (sensor, prevalence, label source, and demographics) that co-vary in our cross-population matrix; we discuss the disentanglement question in Section 5.4 and Section 5.6.

## 5. Discussion

### 5.1. Principal Findings

The experiments yielded three main findings, none of which were expected.

The first is H1. We began the study assuming that the performance of a backbone pretrained on mammography text–image pairs (Mammo-CLIP) or mammography images (Mammo-FM) would surpass that of an ImageNet ResNet-50 by a few AUC points once both went through the same fine-tuning pipeline. It does not. ResNet-50 finishes at 0.867 on the CBIS-DDSM held-out partition. Mammo-CLIP reaches 0.847, and Mammo-FM reaches 0.846. Both gaps survive paired DeLong testing after Benjamini–Hochberg correction (Mammo-CLIP *p_adj* = 0.047, Mammo-FM *p_adj* = 0.031). The baseline wins by roughly 0.02 AUC, and the margin is in the wrong direction from the H1 prediction. DINOv2 and Rad-DINO fare worse still. That said, the dense-breast subgroup tells a different story (see Section 5.3), and rejecting H1 should not be construed as foundation-model pretraining providing no useful information.

The second is H2. The density-aware focal loss, designed to push alpha and gamma away from class frequency toward tissue density, compromised performance on every auxiliary weight setting tested. At w = 0.1, the AUC dropped by 0.031 relative to the baseline. At w = 0.5, the drop grew to 0.061. Calibration degraded at the same time: the ECE doubled from 0.055 to 0.104 to 0.116. The pattern is monotonic with weight, which argues against random instability and toward a systematic interaction between the density-conditioning gradient and the malignancy discrimination objective. Two mechanisms seem plausible. The auxiliary density head may be pulling shared backbone representations in a direction that flattens the malignancy boundary in the feature space, or sufficient noise within the CBIS-DDSM density labels may corrupt the conditioning signal. Separating these issues would require either a relabeling study or an architecture that isolates the density representation from the malignancy pathway.

The third is H3, i.e., the cross-population transfer finding. Taking the best in-distribution model (ResNet-50) and applying it to three external cohorts spanning China (CMMD), India (DMID), and the United Kingdom (MIAS), the AUC dropped by 0.236 to 0.320 points. At a clinical operating point of 95% specificity, sensitivity collapsed from 0.465 in-distribution to between 0.107 and 0.141 across the three external targets. DINOv2 reached 0.504 on CMMD across three seeds, which was statistically indistinguishable from a random classifier (Table 5b). The pairwise transfer tests in Table 5b add a second-order finding to H3: Mammo-CLIP B5 and Mammo-FM significantly outperform ResNet-50 on DMID, and Mammo-CLIP also significantly outperforms ResNet-50 on CMMD; the MIAS comparisons remain directional at *N* = 322. Breast-imaging pretraining therefore appears to provide a measurable but partial transfer benefit for some target populations under our protocol, without eliminating the underlying cross-population drop.

### 5.2. Methodological Implications for Foundation-Model Benchmarks

The divergence between our findings for H1 and the metrics reported in the originating papers carries an implication for how new mammography foundation models should be benchmarked. Mammo-CLIP [8] and Mammo-FM [9] each reported in-distribution gains of 2 to 5 AUC points over strong ResNet baselines in their original evaluations. Under our protocol, the gap runs in the opposite direction. Several factors could explain the divergence, and each has methodological consequences worth naming.

First, the originating papers compared against baselines tuned on different splits and with different augmentation recipes. Our protocol holds those variables fixed, which removes some of the foundation-model advantage. The lesson is that benchmark comparisons across fine-tuning training recipes are not directly comparable, and the field would benefit from publishing fine-tuned baseline AUCs alongside any new foundation-model release. Second, CBIS-DDSM is a relatively clean, film-digitized corpus where a compact discriminative model can extract most of the available signal with limited fine-tuning data. The foundation-model premium may be larger on noisier or more heterogeneous training corpora, and a single in-distribution number cannot resolve this issue. Third, our fine-tuning budget was 10 epochs with a standard cosine-decay schedule. A longer fine-tuning pass with a careful learning-rate search might narrow the gap, although the H1 data suggest it would narrow it in the wrong direction for the foundation models. The dense-breast subgroup result discussed in Section 5.3 sharpens this picture: aggregate AUC obscures the one cell where domain-specific pretraining demonstrably helps.

### 5.3. The Dense-Breast Subgroup Exception

One cell in the H1 picture reverses the directional pattern. Mammo-FM reaches D4 AUC 0.870 against ResNet-50’s 0.842 in the extremely dense bin (*n* = 140). The gap is 0.028, and the confidence intervals are wide at this sample size, so this finding should be interpreted with caution. But the direction is consistent with what mammography-specific pretraining could plausibly offer: a model that has seen a large corpus of dense-tissue mammograms during pretraining may develop feature channels that are more sensitive to malignancy signals buried in dense parenchyma. Whether this survives a larger dense-breast cohort study is an open question, and the clinical stakes are high enough that it is worth pursuing. This is the one subgroup finding that warrants targeted analysis in a follow-on study.

### 5.4. Why the Transfer Gap Is a Clinical Problem

The cross-population drop reported in Section 4.5 is large but not unprecedented. Condon et al. [7] reported AUC 0.83 to 0.76 on the NYU1 model deployed to an Australian cohort and AUC 0.89 to 0.84 on the NYU2 image-encoder variant under matched out-of-the-box evaluation. Our drops of 0.236 to 0.320 are larger, which is plausibly explained by three factors. First, CMMD and DMID skew toward dense breasts more than the Australian cohort, and dense-tissue cancers are systematically harder to detect. Second, our evaluation is image level rather than the exam-level multi-view aggregation used in the NYU work, which tends to inflate the apparent drop on a per-image basis. Third, the prevalence shift is substantially larger: CMMD is roughly 70% positive versus 40% in CBIS-DDSM, whereas the NYU and Australia cohorts exhibit the same low single-digit screening prevalence range.

A second nuance is that the four cohorts differ along several axes simultaneously: X-ray detector technology (film-digitized for CBIS-DDSM and MIAS, full-field digital for CMMD, and mixed digital for DMID); class prevalence (40% vs. 70% vs. 23% vs. 16%); labeling strategy (pathology + BI-RADS vs. biopsy vs. radiology report); and patient demographics (US vs. Chinese vs. Indian vs. UK). Our experimental design cannot cleanly attribute the observed performance drops to sensor shift, population shift, label-noise shift, or prevalence shift individually, because all four sources of distributional change co-vary across the cohorts. Figure 7 illustrates this discrepancy at the pixel-intensity level: the three locally-cached cohorts (CMMD, DMID, and MIAS) have visibly different breast-tissue intensity distributions (mean intensities of 0.40, 0.64, and 0.47, respectively, after shared preprocessing), and the source CBIS-DDSM corpus uses the same film-digitized technology family as MIAS but a different acquisition era. We emphasize that the cohort-intensity comparison in Figure 7 is not by itself proof of sensor-induced domain shift. The distributions clearly differ across cohorts, and this observation is consistent with a sensor-shift contribution to transfer drop, but the comparison does not by itself establish that the sensor axis (rather than, for example, the prevalence or labeling axis) is the dominant driver. The intensity-distribution finding should be interpreted as evidence in support of a sensor-shift interpretation, not as evidence that establishes it. A controlled disentanglement of these factors would require either matched cohorts that vary only along one axis at a time or a prospective study with shared acquisition protocols across populations. We treat this disentanglement as a major direction for follow-on work (Section 5.7) and accordingly soften the absolute strength of the H3 claim in Section 6.

The clinical implications, with that caveat understood, are still immediate. Mammography AI products approved in one national context are increasingly being adopted in others. A US-trained model moving to India faces the DMID scenario in our experiments, where the mean AUC after transfer sits at 0.547, and sensitivity at 95% specificity falls to 0.107. At that level, the model would, based on the evidence presented herein, add little value over a calibrated clinical risk score in the deployed setting. A radiologist deploying the model on a population for which the model was not trained, on this basis, should treat the AI output with substantial skepticism.

The calibration numbers sharpen the concern. Mammo-CLIP on CMMD carries an ECE of 0.403. If a radiology information system displays that model’s output as a probability, it is displaying a number that is on average 40 percentage points off from empirical prevalence. The displayed 30% probability of malignancy may correspond to an actual rate closer to 70%, or to 5%, depending on the direction of the miscalibration. Temperature scaling can correct this on a local hold-out, but only if the deploying institution has the labeled data and the workflow to run the calibration step. Many lower-resource settings do not.

The Mammo-CLIP and Mammo-FM results at transfer are slightly better than those of ResNet-50 at some targets, particularly on DMID and MIAS. The absolute level is still far below any clinical threshold. The honest framing is that foundation models reduce the transfer gap modestly at some targets but do not eliminate it, and the remaining gap is large enough to make cross-population deployment unsafe without local validation.

### 5.5. Practical Recommendations for Clinical Deployment

Five concrete recommendations follow from the results, addressed to research teams, deploying institutions, and regulators in turn.

For research teams reporting new mammography foundation models, in-distribution AUC alone is insufficient evidence of clinical utility. The H1 result shows that controlled fine-tuning with shared splits can reverse a reported foundation-model advantage, and the per-subgroup analysis in Table 4 shows that aggregate AUC obscures the cells where domain-specific pretraining actually helps. Future foundation-model releases should include subgroup-stratified evaluation against fine-tuned baselines and on at least one out-of-distribution cohort.

For deploying institutions, AUC measured on the training population is not a reliable proxy for in-clinic performance. The drops we observed at transfer, 0.2 to 0.3 AUC points and 0.20 to 0.36 absolute reduction in sensitivity at 95% specificity, are large enough to convert a diagnostic-quality tool into something that adds no value over a naïve threshold. Subgroup-stratified external validation, covering at minimum the acquisition protocol and population demographics of the intended deployment setting, should occur before any clinical use.

For deploying institutions, recalibration on a local hold-out is not optional once a model leaves its training population. The temperature-scaling experiment in Table 7 shows that source-fitted recalibration delivers modest improvement (Δ ECE −0.01 to −0.05) but does not restore clinically acceptable calibration on its own. A two-stage recalibration pathway is preferable: temperature scaling on source validation as a baseline step, then local labeled recalibration (isotonic regression or conformal prediction) once a small target-domain calibration set is available.

For regulators reviewing AI-assisted mammography systems, evidence requirements should include subgroup-stratified external validation, a local recalibration plan, and per-population performance reporting. The current frameworks accept in-distribution AUC as the primary outcome, which our findings show is insufficient for cross-population deployment safety. The clinical risk pattern we document, in which sensitivity at the operating point can fall to 0.11 in a population for which the model was not trained, is exactly the failure mode that post-market surveillance is poorly equipped to detect.

For dense-breast screening specifically, the Mammo-FM D4 result, while modest in magnitude (0.028 AUC over ResNet-50 at *n* = 140), points to a clinically meaningful direction. The field should design dense-breast-targeted evaluations rather than relying on aggregate AUC and should consider mammography-specific pretrained backbones for the dense-tissue cohort even when aggregate metrics favor ImageNet baselines.

### 5.6. Limitations

Five limits sit at the front of the queue.

The first is the absence of per-density subgroup analysis on external cohorts. As described in Section 3.5, CMMD contains no density labels in its metadata, while MIAS and DMID use the legacy three-class fatty/glandular/dense tag rather than the four-class BI-RADS A–D scheme. We do not extend per-density subgroup AUC reporting to the external cohorts in this study, and we treat radiologist-confirmed BI-RADS rereading of those cohorts (or a high-quality auxiliary classifier externally validated against a public cohort that already ships native BI-RADS, such as RSNA Screening) as planned follow-on work. The Mammo-FM D4 advantage observation in Table 4 is therefore restricted to CBIS-DDSM, where BI-RADS density labels are present in the original metadata and the per-density subgroup analysis carries no auxiliary-labeling caveat.

The second is the MIAS cohort size. MIAS has 322 images, and confidence intervals on transfer AUC at this sample size are wide (roughly ±0.09 at 95% coverage). The MIAS transfer results should be interpreted as directionally indicative, not as precisely estimated effects.

The third is DINOv2 learning-rate sensitivity. The model collapsed at lr = 1 × 10^−4^ and lr = 5 × 10^−5^ (AUC near 0.59 to 0.60 with high variance) before recovering at lr = 1 × 10^−5^. The lr = 1 × 10^−5^ result still shows SD = 0.028 across seeds, which is notably wider than any other backbone. This suggests that the DINOv2 fine-tuning optimum is sharper and harder to find than for the convolutional models.

The fourth is single-source training and the resulting compound shift at transfer. All in-distribution training used CBIS-DDSM as the sole source. The three external evaluation cohorts differ from CBIS-DDSM along at least four axes simultaneously: X-ray detector technology, malignancy prevalence, label source, and patient demographics (see Section 5.4). Our experimental design therefore cannot isolate the impacts of these axes on the observed performance drops. Readers should treat every transfer result in Table 5 (and the per-target significance results in Table 5b,c) as a compound-shift observation, not as evidence for a specific causal mechanism. A bidirectional cross-population study with separate models trained on each cohort, or a matched-cohort study where only one axis varies at a time, would enable controlled disentanglement; both are planned follow-on work in Section 5.7. We have therefore made the compound-shift framing explicit here, in the Section 4.5 narrative, and in the Conclusions.

The fifth is single-GPU compute. All experiments ran on one RTX 5070 Ti. The hyperparameter search is bounded by what fits a 12 to 15 day window, and a more thorough search over the DAF (alpha_d, gamma_d) space might find a less destructive configuration, although the monotonic AUC degradation argues against a simple fix from a wider search alone.

The sixth is the broader question of how to handle seed-to-seed variability in significance testing on small medical imaging cohorts. The conventional stacked-predictions paired DeLong test (used in Table 5b) is fast and aligns with the medical imaging AI literature default, but it fails to detect the within-patient correlation across seeds. The patient-level cluster bootstrap (added in Table 2 and Table 5) and the per-seed Stouffer meta-analysis (added in Table 5c) are two more conservative alternatives that we report alongside the stacked test. A hierarchical bootstrap that draws first at the patient level and then at the seed level, or a fully Bayesian hierarchical model treating seed as a random effect, would be a methodologically tighter framework than any of the three we report. We do not implement these here because they would change the inferential paradigm of the entire benchmark, but we flag this option as a useful direction for a bidirectional follow-on study. The general lesson, in our view, is that seed variability deserves to be treated as an explicit modeling decision in mammography AI benchmarks, not as a nuisance averaged into a single mean.

### 5.7. Future Work

We identify five directions for future work. The first is a proper dense-breast cohort study. The D4 Mammo-FM signal in Section 4.4 is intriguing but underpowered at *n* = 140. An INbreast extension or a prospective clinical collection from a breast imaging center would give the statistical power to resolve whether mammography pretraining carries a genuine dense-tissue advantage.

The second is replacing the density-aware focal loss with a subgroup-balanced objective that does not interfere with focal calibration. Group DRO (Sagawa et al., ICLR 2020 [27]) minimizes worst-subgroup loss and has demonstrated +17.4 worst-subgroup F1 over focal baselines on long-tail medical classification tasks. Just Train Twice (JTT, Liu et al., ICML 2021 [28]) achieves comparable worst-subgroup recovery without group annotations, which is attractive given the noisy auto-derived density labels on three of our four cohorts. A direct ablation against our DAF result would clarify whether the failure is specific to density-conditioned focal reweighting or general to subgroup-conditioned loss reweighting on top of focal calibration.

The third is a bidirectional cross-population training design. Training separate models on each of the four cohorts and evaluating across all pairs would produce a complete 4 by 4 transfer matrix and disentangle population-specific factors from scanner-specific factors.

The fourth is calibration and prior-shift correction as first-class objectives. The transfer ECE numbers in Section 4.6 indicate that source-fitted temperature scaling delivers only modest improvement at transfer, and the prevalence shift between CBIS (40% positive) and CMMD (70%), DMID (23%), or MIAS (16%) is large enough to confound AUC interpretation. The Saerens EM prior-shift correction (Saerens et al., Neural Computation 2002 [25]) and bias-corrected temperature scaling under covariate shift (BBSC and RLLS variants documented in subsequent work) restore threshold-based metrics like sensitivity at 95% specificity under prevalence shift without retraining and would be a natural follow-on combined with multi-domain temperature scaling.

The fifth is federated learning. The cross-population gap exists partly because pooling raw DICOM across borders faces legal and logistical barriers. Federated learning with gradient exchange rather than data sharing would expand the cross-population training matrix without these constraints.

## 6. Conclusions

We deliver three contributions: a controlled head-to-head benchmark of five backbone architectures (ResNet-50, DINOv2-B14, Rad-DINO, Mammo-CLIP B5, and Mammo-FM) on a patient-disjoint CBIS-DDSM split, a systematic ablation of a density-aware focal loss across three auxiliary weights, and the first cross-population transfer matrix covering CBIS-DDSM, CMMD, DMID, and MIAS under one shared fine-tuning protocol. All three were evaluated with paired DeLong testing and Benjamini–Hochberg FDR correction.

The benchmark shows that under controlled fine-tuning with shared splits, mammography-specific pretraining did not, in our protocol, deliver the in-distribution AUC premium reported in the originating Mammo-CLIP and Mammo-FM papers. ResNet-50 leads at AUC 0.867, with Mammo-CLIP at 0.847 and Mammo-FM at 0.846, and all gaps are significant after FDR correction. The result has two interpretations, and we report both. At the aggregate level, ImageNet-pretrained ResNet-50 holds its ground when given the same fine-tuning training recipe. At the subgroup level, Mammo-FM outperforms ResNet-50 in the extremely dense bin (D4 AUC 0.870 vs. 0.842), although the 95% CIs of the two models overlap heavily at the D4 sample size of *n* = 140, so this cell should be interpreted as a directional signal worth following up rather than a statistically supported advantage. The methodological implication for the field is that aggregate AUC obscures subgroup signals worth quantifying and that benchmark comparisons across fine-tuning training recipes are not directly comparable.

The density-aware focal loss ablation reveals an adverse interaction between subgroup-conditional loss reweighting and end-to-end backbone fine-tuning at every weight tested. The AUC dropped 0.031 to 0.061 points and the ECE doubled, showing a monotonic relationship with auxiliary weight. The ablation experiment rules out a simple loss-weight tuning fix and points the next round of subgroup-aware loss design toward architectural separation of the conditioning pathway or cleaner radiologist-confirmed density labels.

The cross-population transfer matrix is where the clinical message lands hardest, with the caveat that the four cohorts vary along multiple axes (sensor technology, prevalence, label source, and population demographics) and our design cannot disentangle them (Section 5.4). Every model, at every target population, lost more than 0.165 AUC points relative to its in-distribution performance, and sensitivity at 95% specificity dropped from 0.31 to 0.47 in-distribution to 0.11 to 0.22 across the three external targets. The pairwise tests in Table 5b add nuance: Mammo-CLIP and Mammo-FM significantly outperform ResNet-50 on DMID and (for Mammo-CLIP) CMMD, suggesting that breast-imaging pretraining provides a measurable transfer benefit for some target populations under our protocol. The MIAS comparisons remain directional only at *N* = 322. The absolute level for every backbone remains substantially below any clinical threshold, and calibration deteriorates from in-distribution ECE 0.055 to ECE 0.40 or worse at transfer.

The practical message for clinical translation is concrete. Mammography AI products approved in one national context cannot be safely deployed in another without subgroup-stratified external validation and local recalibration. Regulatory frameworks should treat both as prerequisites rather than as post-market afterthoughts. For the research community, the open benchmark protocol and four-population evaluation pipeline released with this paper give a defensible baseline for testing the next generation of mammography foundation models, domain adaptation methods, and federated training approaches as they become available.

Reporting controlled null results on a shared protocol is, in our view, a necessary part of building a reliable evidence base for clinical medical imaging AI. We frame the negative H1 and H2 findings and the H3 transfer drops moderated by per-target significance testing in that spirit. All model weights, training code, the density-aware loss implementation, the evaluation pipeline (including the new patient-level cluster bootstrap and transfer-target paired DeLong scripts), and the per-cohort intensity-distribution analysis are released under MIT license at https://github.com/sommossgl/paper-03-density-aware-mammography (accessed on 9 June 2026). We particularly invite other groups to extend the cross-population matrix to cohorts to which we did not have access, including Southeast Asian, Latin American, and African mammography collections where the coverage gap in the published literature is widest.

## Figures and Tables

**Table 1:** Dataset characteristics and validation set sizes used in this study.

Dataset	Country	*N* (Val)	Positive Rate	Acquisition	Label Source
CBIS-DDSM	USA	714	40%	Film-digitized	Pathology + BI-RADS
CMMD	China	1032	70%	Full-field digital	Biopsy
DMID	India	509	23%	Mixed digital	Radiology report
MIAS	UK	322	16%	Film-digitized	Radiology report

**Table 2:** In-distribution performance on CBIS-DDSM validation set (*N* = 714, 3 seeds each). AUC is reported as mean ± standard deviation across seeds. Both the standard DeLong 95% CI (computed on stacked predictions) and a patient-level cluster bootstrap 95% CI (5000 iterations, resampling patients with replacement) are reported. Sens@95Spec is sensitivity at 95% specificity. Brier and ECE are calibration metrics; lower is better. The Sens@95Spec entry is omitted for DAF variants because the density-conditional auxiliary loss complicates threshold selection at a fixed specificity (see footnote (a)). (a) Sensitivity at 95% specificity is omitted for the DAF variants because the density-conditional auxiliary loss complicates threshold selection at a fixed specificity. The DAF loss reweights samples by BI-RADS density bin, which shifts the score distribution non-uniformly across the operating range and makes the 95% specificity threshold no longer directly comparable across the DAF rows and the baseline rows. See Section 3.3 and Section 4.3 for details.

Model	AUC Mean ± SD	DeLong 95% CI	Bootstrap 95% CI	Sens@95%Spec	Brier	ECE
ResNet-50 (baseline)	0.867 ± 0.006	[0.860, 0.871]	[0.842, 0.889]	0.465 ± 0.023	0.147	0.055
Mammo-CLIP B5	0.847 ± 0.007	[0.841, 0.855]	[0.817, 0.868]	0.413 ± 0.046	0.166	0.105
Mammo-FM (BatmanLab)	0.846 ± 0.006	[0.841, 0.852]	[0.815, 0.865]	0.414 ± 0.015	0.165	0.096
DINOv2-B14 (lr = 1 × 10^−5^)	0.813 ± 0.028	[0.781, 0.830]	[0.775, 0.830]	0.310 ± 0.057	0.178	0.083
Rad-DINO	0.703 ± 0.047	[0.674, 0.758]	[0.671, 0.727]	0.140 ± 0.063	0.216	0.060
ResNet-50 + DAF (w = 0.1)	0.836 ± 0.009	[0.829, 0.846]	[0.805, 0.858]	— (a)	0.174	0.114
ResNet-50 + DAF (w = 0.3)	0.819 ± 0.008	[0.810, 0.826]	[0.787, 0.844]	— (a)	0.184	0.116
ResNet-50 + DAF (w = 0.5)	0.806 ± 0.008	[0.797, 0.812]	[0.773, 0.832]	— (a)	0.188	0.104

**Table 3:** Paired DeLong significance test: each model versus ResNet-50 baseline (predictions stacked across 3 seeds, *n* = 2142). All *p*-values are upper bounds (independent-SE approximation), corrected with Benjamini–Hochberg FDR.

Model	AUC	Δ vs. Baseline	*p* (DeLong)	*p_adj* (BH-FDR)
Mammo-CLIP B5	0.8438	−0.023	0.047	0.047
Mammo-FM (BatmanLab)	0.8411	−0.025	0.026	0.031
DINOv2-B14 (lr = 1 × 10^−5^)	0.8043	−0.062	<0.001	<0.001
Rad-DINO	0.6987	−0.168	<0.001	<0.001
ResNet-50 + DAF (w = 0.1)	0.8332	−0.033	0.004	0.006
ResNet-50 + DAF (w = 0.3)	0.8161	−0.050	<0.001	<0.001
ResNet-50 + DAF (w = 0.5)	0.8037	−0.063	<0.001	<0.001

**Table 4:** Per-density subgroup AUC on CBIS-DDSM validation, averaged across 3 seeds (point estimate; 95% DeLong CIs are reported in Figure 3 error bars). D1 = fatty, D2 = scattered, D3 = heterogeneously dense, D4 = extremely dense. The Mammo-FM D4 advantage of +0.028 over ResNet-50 sits within the overlapping 95% CIs of the two models ([0.818, 0.892] for Mammo-FM vs. [0.798, 0.881] for ResNet-50), so this cell should be read as directional only, not as a significant difference.

Model	D1 (*n* = 100)	D2 (*n* = 264)	D3 (*n* = 210)	D4 (*n* = 140)
ResNet-50 (baseline)	0.912	0.876	0.860	0.842
Mammo-CLIP B5	0.874	0.879	0.821	0.803
Mammo-FM (BatmanLab)	0.836	0.871	0.812	0.870
DINOv2-B14 (lr = 1 × 10^−5^)	0.869	0.847	0.780	0.755

**Table 5:** (**a**) Cross-population transfer performance: CBIS-trained models evaluated on three external cohorts. AUC values are mean ± SD across three seeds, with patient-level cluster bootstrap 95% CIs (5000 iterations) reported separately. Drop is the difference relative to the source CBIS-DDSM AUC. Sens@95Spec is sensitivity at 95% specificity (stacked across seeds). Rad-DINO is omitted from this table because its in-distribution AUC of 0.703 falls substantially below the threshold typically required to claim a functional binary classifier on CBIS-DDSM (see Section 4.2); evaluating its transfer would not yield scientifically meaningful generalization data. Pairwise statistical comparisons against ResNet-50 at each transfer target are reported in (**b**). (**b**) Paired DeLong significance test for each foundation backbone versus the ResNet-50 baseline at each transfer target. *p*-values are conservative upper bounds from the independent-SE approximation (see Section 3.6), corrected with Benjamini–Hochberg FDR across all nine comparisons. Four of nine comparisons reach statistical significance; the remaining five (Mammo-FM on CMMD, DINOv2 on DMID, and all three comparisons on MIAS) cannot be distinguished from the baseline at α = 0.05, which is partly explained by the small MIAS cohort (*N* = 322), where bootstrap CIs span roughly ±0.08 AUC. (**c**) Per-seed paired DeLong + Stouffer meta-analysis at transfer targets. Added during peer-review revision to address the concern that the stacked-prediction DeLong test in (**b**) may inflate the effective sample size. For each model × target cell, we ran a paired DeLong z-test separately for each seed (three independent tests; no stacking), then combined the per-seed z-statistics via Stouffer’s method z_pool = sum(z_i)/sqrt(k). The pooled *p*-value is BH-FDR-corrected across all nine transfer comparisons. I^2^ is the Cochran heterogeneity statistic across the three seeds (0 percent = perfect agreement, higher = stronger disagreement). The pooled test is more conservative than (**b**) (the independence assumption no longer inflates sample size) and is reported here as the primary inference for transfer comparisons; (**b**) is retained for direct comparability with the stacked-prediction convention used in much of the medical imaging AI literature. Footer notes: ✓ indicates a statistically significant difference at α = 0.05 after Benjamini–Hochberg FDR correction; ns indicates not significant at the same threshold. Bold formatting flags the four-cell significance pattern.

(**a**)							
**Model**	**Target**	**AUC (Transfer)**	**Bootstrap 95% CI**	**AUC Drop**		**Sens@95%Spec**	
ResNet-50	CMMD (China)	0.568 ± 0.020	[0.532, 0.590]	−0.299		0.115	
Mammo-CLIP B5	CMMD (China)	0.612 ± 0.024	[0.579, 0.642]	−0.235		0.156	
Mammo-FM	CMMD (China)	0.561 ± 0.034	[0.527, 0.591]	−0.286		0.119	
DINOv2-B14	CMMD (China)	0.504 ± 0.019	[0.467, 0.528]	−0.309		0.108	
ResNet-50	DMID (India)	0.547 ± 0.029	[0.487, 0.601]	−0.320		0.107	
Mammo-CLIP B5	DMID (India)	0.644 ± 0.059	[0.583, 0.679]	−0.203		0.178	
Mammo-FM	DMID (India)	0.612 ± 0.048	[0.559, 0.664]	−0.234		0.175	
DINOv2-B14	DMID (India)	0.565 ± 0.092	[0.515, 0.611]	−0.248		0.153	
ResNet-50	MIAS (UK)	0.630 ± 0.019	[0.542, 0.701]	−0.236		0.141	
Mammo-CLIP B5	MIAS (UK)	0.669 ± 0.036	[0.586, 0.736]	−0.178		0.186	
Mammo-FM	MIAS (UK)	0.681 ± 0.020	[0.605, 0.755]	−0.165		0.224	
DINOv2-B14	MIAS (UK)	0.618 ± 0.071	[0.536, 0.669]	−0.195		0.186	
(**b**)							
**Target**	**Model**	**AUC**	**Δ vs. ResNet-50**	***p*** **(DeLong)**	***p_adj*** **(BH-FDR)**		**Sig?**
CMMD (China)	Mammo-CLIP B5	0.6104	+0.049	0.0034	0.0101		✓
CMMD (China)	Mammo-FM	0.5600	−0.001	0.9365	0.9365		ns
CMMD (China)	DINOv2-B14	0.4977	−0.064	0.0002	0.0020		✓
DMID (India)	Mammo-CLIP B5	0.6319	+0.087	0.0005	0.0023		✓
DMID (India)	Mammo-FM	0.6119	+0.067	0.0090	0.0203		✓
DMID (India)	DINOv2-B14	0.5632	+0.018	0.4761	0.6121		ns
MIAS (UK)	Mammo-CLIP B5	0.6621	+0.040	0.2675	0.4013		ns
MIAS (UK)	Mammo-FM	0.6799	+0.058	0.1131	0.2035		ns
MIAS (UK)	DINOv2-B14	0.6045	−0.018	0.6314	0.7103		ns
(**c**)							
**Target**	**Model**	**Mean Δ**	**Pooled z**	**Pooled *p***	***p_adj*** **(BH-FDR)**	**I^2^**	**Sig?**
CMMD (China)	Mammo-CLIP B5	+0.045	+2.71	0.0068	0.019	55%	✓
CMMD (China)	Mammo-FM	−0.007	−0.41	0.6833	0.769	0%	ns
CMMD (China)	DINOv2-B14	−0.064	−3.77	0.0002	0.001	19%	✓
DMID (India)	Mammo-CLIP B5	+0.098	+3.99	0.0001	0.001	74%	✓
DMID (India)	Mammo-FM	+0.066	+2.64	0.0084	0.019	68%	✓
DMID (India)	DINOv2-B14	+0.019	+0.68	0.4978	0.640	82%	ns
MIAS (UK)	Mammo-CLIP B5	+0.039	+1.08	0.2800	0.420	0%	ns
MIAS (UK)	Mammo-FM	+0.051	+1.41	0.1583	0.285	0%	ns
MIAS (UK)	DINOv2-B14	−0.012	−0.29	0.7687	0.769	24%	ns

**Table 6:** Expected calibration error (ECE) in-distribution and at three transfer targets. Lower values indicate better-calibrated probability estimates.

Model	CBIS (USA)	CMMD (China)	DMID (India)	MIAS (UK)
ResNet-50	0.055	0.186	0.253	0.246
Mammo-CLIP B5	0.105	0.403	0.228	0.360
Mammo-FM	0.096	0.319	0.306	0.360
DINOv2-B14	0.083	0.237	0.374	0.446

**Table 7:** Temperature scaling recalibration: temperature (T) fitted on CBIS-DDSM validation predictions and applied to each transfer target. ECE before and after scaling reported with absolute improvement.

Model	T	Target	ECE Before	ECE After	Δ ECE
ResNet-50	1.38	CMMD	0.182	0.172	−0.010
ResNet-50	1.38	DMID	0.246	0.237	−0.009
ResNet-50	1.38	MIAS	0.242	0.228	−0.014
Mammo-CLIP B5	2.11	CMMD	0.400	0.350	−0.049
Mammo-CLIP B5	2.11	DMID	0.219	0.197	−0.022
Mammo-CLIP B5	2.11	MIAS	0.350	0.335	−0.015
Mammo-FM	1.70	CMMD	0.309	0.278	−0.031
Mammo-FM	1.70	DMID	0.303	0.288	−0.014
Mammo-FM	1.70	MIAS	0.355	0.335	−0.020
DINOv2-B14	1.58	CMMD	0.237	0.214	−0.023
DINOv2-B14	1.58	DMID	0.374	0.335	−0.039
DINOv2-B14	1.58	MIAS	0.445	0.410	−0.035

## Data Availability

CBIS-DDSM is available from The Cancer Imaging Archive at https://www.cancerimagingarchive.net/collection/cbis-ddsm (accessed on 9 June 2026). CMMD is available from The Cancer Imaging Archive at https://www.cancerimagingarchive.net/collection/cmmd (accessed on 9 June 2026). DMID is available from the original publishers as referenced. MIAS is available at https://www.mammoimage.org/databases/ (accessed on 9 June 2026). All training code, model weights, and analysis scripts will be released at https://github.com/sommossgl/paper-03-density-aware-mammography (accessed on 9 June 2026) under MIT license upon publication.
